# A longitudinal plasma lipidomics dataset from children who developed islet autoimmunity and type 1 diabetes

**DOI:** 10.1038/sdata.2018.250

**Published:** 2018-11-13

**Authors:** Santosh Lamichhane, Linda Ahonen, Thomas Sparholt Dyrlund, Heli Siljander, Heikki Hyöty, Jorma Ilonen, Jorma Toppari, Riitta Veijola, Tuulia Hyötyläinen, Mikael Knip, Matej Orešič

**Affiliations:** 1 Turku Centre for Biotechnology, University of Turku and Åbo Akademi University, Turku 20520, Finland; 2 Steno Diabetes Center Copenhagen, 2820 Gentofte, Denmark; 3 Children’s Hospital, University of Helsinki and Helsinki University Hospital, 00290 Helsinki, Finland; 4 Research Program Unit, Diabetes and Obesity, University of Helsinki, 00290 Helsinki, Finland; 5 Faculty of Medicine and Life Sciences, University of Tampere, Tampere, Finland; 6 Fimlab Laboratories, Pirkanmaa Hospital District, Tampere, Finland; 7 Immunogenetics Laboratory, Institute of Biomedicine, University of Turku, Turku, Finland; 8 Clinical Microbiology, Turku University Hospital, Turku, Finland; 9 Institute of Biomedicine, Centre for Integrative Physiology and Pharmacology, University of Turku, Turku, Finland; 10 Department of Pediatrics, Turku University Hospital, Turku, Finland; 11 Department of Paediatrics, PEDEGO Research Unit, Medical Research Centre, University of Oulu, Oulu, Finland; 12 Department of Children and Adolescents, Oulu University Hospital, Oulu, Finland; 13 Department of Women’s and Children’s Health, Karolinska Institutet, Stockholm, Sweden; 14 Department of Chemistry, Örebro University, 702 81 Örebro, Sweden; 15 Tampere Center for Child Health Research, Tampere University Hospital, Tampere, Finland; 16 Folkhälsan Research Center, Helsinki, Finland; 17 School of Medical Sciences, Örebro University, 702 81 Örebro, Sweden

**Keywords:** Metabolomics, Type 1 diabetes, Lipidomics

## Abstract

Early prediction and prevention of type 1 diabetes (T1D) are currently unmet medical needs. Previous metabolomics studies suggest that children who develop T1D are characterised by a distinct metabolic profile already detectable during infancy, prior to the onset of islet autoimmunity. However, the specificity of persistent metabolic disturbances in relation T1D development has not yet been established. Here, we report a longitudinal plasma lipidomics dataset from (1) 40 children who progressed to T1D during follow-up, (2) 40 children who developed single islet autoantibody but did not develop T1D and (3) 40 matched controls (6 time points: 3, 6, 12, 18, 24 and 36 months of age). This dataset may help other researchers in studying age-dependent progression of islet autoimmunity and T1D as well as of the age-dependence of lipidomic profiles in general. Alternatively, this dataset could more broadly used for the development of methods for the analysis of longitudinal multivariate data.

## Background & Summary

Type 1 diabetes (T1D) is a chronic, autoimmune disease caused by progressive loss of insulin-secreting capacity due to the selective death of beta cells in the islets of Langerhans, of which there are more than one million in the human pancreas^1^. The age of onset is usually between 5–15 years, but, in recent years, many children before 5 years of age are being affected^2^. Although approximately 80% of subjects with T1D carry defined risk-associated genotypes at the human leukocyte antigen (HLA) locus, only 3–7% of the carriers of such genetic risk markers go on to develop overt disease. Seroconversion to islet autoantibody positivity is the first detectable signal demonstrating initiation of autoimmunity and risk of progression towards diabetes^3,4^. However, whilst seroconversion to autoantibody positivity precedes clinical disease by months to years, the point at which seroconversion occurs may already be too late for therapeutic approaches aimed at preventing progression to overt diabetes.

Previous metabolomic studies suggest that children who develop islet autoimmunity and/or T1D are characterised by specific metabolic disturbances prior to the first appearance of islet autoantibodies^5–7^. Metabolic profiling may thus be of clinical relevance by providing a complimentary tool for estimating risk of progression to T1D. However, the underlying causes of these early metabolic disturbances and their link to disease progression are still largely unknown. It is also not yet established if the observed, persistent metabolic changes are specifically associated with the progression to T1D, or more broadly, if they are associated with progression to islet autoimmunity and irrespective of disease outcome.

**Figure 1:**
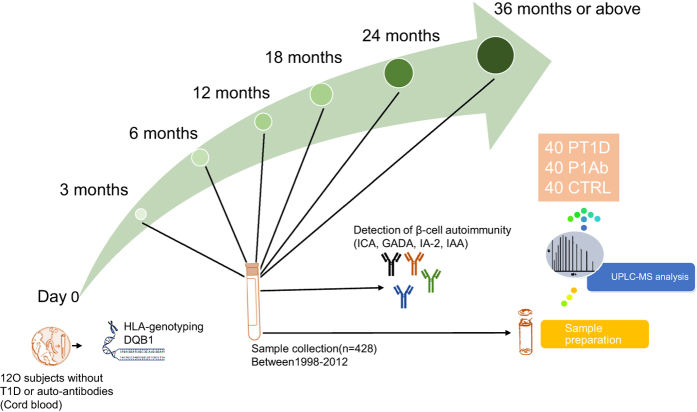
An overview of the study design. In this study, newborn infants at the University Hospital of Tampere were screened for increased genetic risk for T1D. This study includes three study groups: (1) 40 T1D (PT1D) progressors, (2) 40 who developed at least a single Ab during the follow-up but did not progress to T1D (P1Ab), and (3) 40 controls (CTRL) who remained autoantibody negative during the follow-up. The three study groups were matched by HLA-associated diabetes risk, gender and period of birth. For each child, up to six longitudinal samples were obtained corresponding to the ages of 3, 6, 12, 18, 24 and 36 (or above) months. Abbreviations: islet cell antibodies (ICA), insulin autoantibodies (IAA), islet antigen 2 (IA-2) autoantibodies, and glutamic acid decarboxylase autoantibodies (GADA).

Here we provide a longitudinal plasma lipidomics dataset obtained from children participating in a prospective, birth cohort Type 1 Diabetes Prediction and Prevention study (DIPP). Three study groups were examined: children who (1) progressed to T1D (PT1D), (2) developed at least a single islet autoantibody (Ab) during follow-up but did not progress to T1D (P1Ab), and (3) controls (CTR) who remained autoantibody negative and healthy during the follow-up until 15 years of age. We analysed 428 plasma samples from 120 children (40 PT1D, 40 P1Ab and 40 CTR). The samples were collected up to six different time points corresponding to the ages of 3, 6, 12, 18, 24, and 36 (or above) months (Fig. 1). These age groups were selected with the objective of understanding the changes in lipidomic profile preceding prior to overt T1D. We performed untargeted lipidomics using ultra-high-performance liquid chromatography combined with quadrupole time-of-flight mass spectrometry (UHPLC-QTOF-MS). Both raw and pre-processed datasets were deposited in the MetaboLights repository (Data Citation 1). Along with the aforementioned lipidomic data, we provide information on the type of islet autoantibodies observed in this longitudinal setting. In this study, sphingomyelins (SMs) were found to be persistently downregulated in PT1D as compared to the P1Ab and CTR groups^8^. Triacylglycerols (TGs) and phosphatidylcholines (PCs) were mainly downregulated in PT1D as compared to P1Ab at the age of 3 months. These results suggest that distinct lipidomic signatures characterise children who progressed to islet autoimmunity or clinical T1D. Lipidomic profiling may thus be helpful in the identification of at-risk children before the initiation of autoimmunity.

This data descriptor is one of the first longitudinal lipidomic datasets allowing the investigation of progression to islet autoimmunity/T1D during the early prodromal phases of disease development. Considering the longitudinal study design, this clinical dataset may have many uses. Firstly, it may assist other researchers in their studies of early pathogenesis of T1D and, potentially, other immune-mediated inflammatory diseases. It may also allow other researchers to study the age-dependent progression of lipidomic profiles during infancy. Finally, the dataset has great potential to be used in the development and testing of algorithms for the analysis of multivariate longitudinal/prospective data.

## Methods

These methods are expanded versions of descriptions in our related work^8^.

### Study design

The plasma samples for lipidomics analysis were obtained from the Finnish Type 1 Diabetes Prevention and Prediction (DIPP) study^9^. The DIPP study has screened more than 220,000 newborn infants for HLA-conferred susceptibility to T1D at three university hospitals in Finland: Turku, Tampere and Oulu^10^. Over 25,000 infants were identified as having an increased genetic risk and approximately 17,000 families joined the follow-up study, which involves regular study center visits (with an interval of 3–6 months). The Ethics and Research Committee of the participating Universities and Hospitals approved the study protocol. The study was conducted according to the guidelines in the Declaration of Helsinki. All families provided written informed consent for participation in the study. At every visit, blood samples were collected to measure the titre of T1D-associated islet autoantibodies. Non-fasting blood samples were collected into sodium citrate tubes. Plasma was separated within 30 min of collection by centrifugation at 1600g for 20 min at room temperature, aliquoted, and stored at −80 °C until analysed. Now, 1,663 of those children (9.8%) have seroconverted to positivity for one autoantibody during the follow-up, 808 (4.8%) have developed multiple autoantibodies, and 510 (3.1%) have progressed to clinical T1D.

The 120 infants included in the current data descriptor were selected from a subset of DIPP children from the city of Tampere. Up to six longitudinal samples per child were collected between 1998 and 2012, corresponding to the ages of 3, 6, 12, 18, 24 and 36 months (Fig. 1, Table 1). The details of the ages and selected characteristics of the current study subjects are given in the metadata (Data Citation 1). The three study groups were matched by HLA-associated diabetes risk, gender and the period of birth. In total, 428 plasma samples were selected and analysed for this study.

### HLA genotyping

HLA-conferred susceptibility to T1D was analysed using cord blood samples as previously described^11^. Briefly, the HLA-genotyping was performed with a time-resolved fluorometry-based assay for four alleles using lanthanide chelate labeled, sequence-specific oligonucleotide probes detecting the DQB1^∗^02, DQB1^∗^03:01, DQB1^∗^03:02 and DQB1^∗^06:02/3 alleles^12^. The carriers of the genotype DQB1^∗^02/DQB1^∗^03:02 or DQB1^∗^03:02/x genotypes (here x ≠ DQB1^∗^02, DQB1^∗^03:01, DQB1^∗^06:02, or DQB1^∗^06:03 alleles) were categorised into the T1D risk group and recruited for the DIPP follow-up program.

### Detection of beta-cell autoimmunity

The participants with HLA-conferred genetic susceptibility were monitored for the appearance of T1D-associated autoantibodies: islet cell antibodies (ICA), insulin autoantibodies (IAA), islet antigen 2 autoantibodies (IA-2A), and glutamic acid decarboxylase autoantibodies (GADA). These autoantibodies were measured in the Diabetes Research Laboratory (University of Oulu) from the plasma samples taken at each follow-up visit^13^. ICA were detected with the use of indirect immunofluorescence, whereas the other three autoantibodies were quantified with the use of specific radiobinding assays^14^. We used cutoff limits for positivity of 2.5 Juvenile Diabetes Foundation (JDF) units for ICA, 3.48 relative units (RU) for IAA, 5.36 RU for GADA, and 0.43 RU for IA-2A. The disease sensitivity and specificity of the assay for ICA were 100% and 98%, respectively, in the fourth round of the international workshops on standardisation of the ICA assay. According to the Diabetes Autoantibody standardisation Program (DASP) and Islet Autoantibody standardisation Program (IASP) workshop results in 2010–2015, disease sensitivities for the IAA, GADA and IA-2A radio binding assays were 36–62%, 64–88% and 62–72%, respectively. The corresponding disease specificities were 94–98%, 94–99% and 93–100%, respectively. The metadata in the Metabolights contains information on the types of autoantibodies detected in the longitudinal plasma samples (Data Citation 1).

### Sample preparation and UHPLC-MS analysis

The solvents and eluent modifiers used in this work were purchased from Sigma-Aldrich (Steinheim, Germany): HPLC-grade chloroform, LC-MS grade acetonitrile (ACN), isopropanol (IPA), water (H_2_O), methanol (MeOH), ammonium acetate (NH_4_Ac), analytical grade formic acid (HCOOH) and sodium chloride (NaCl).

The following internal standards were purchased for quality control (QC) and calibration purposes: 1,2-diheptadecanoyl-*sn*-glycero-3-phosphoethanolamine (PE(17:0/17:0)), N-heptadecanoyl-D-*erythro*-sphingosylphosphorylcholine (SM(d18:1/17:0)), N-heptadecanoyl-D-*erythro*-sphingosine (Cer(d18:1/17:0)), 1,2-diheptadecanoyl-*sn*-glycero-3-phosphocholine (PC(17:0/17:0)), 1-heptadecanoyl-2-hydroxy-*sn*-glycero-3-phosphocholine (LPC(17:0)) and 1-palmitoyl-d31-2-oleoyl-*sn*-glycero-3-phosphocholine (PC(16:0/d31/18:1)) from Avanti Polar Lipids, Inc. (Alabaster, Alabama, USA), 1,2-Dimyristoyl-*sn*-glycero-3-phospho(choline-d_13_) (PC(14:0/d13)) from Sigma-Aldrich and Tripalmitin-1,1,1-13C3 (TG(16:0/16:0/16:0)-13C3) and Trioctanoin-1,1,1-13C3 (TG(8:0/8:0/8:0)-13C3) from Larodan (Solna, Sweden). Stock solutions (1.0 mg mL^−1^) of the internal standards were prepared by dissolving the analytes in CHCl_3_:MeOH (2:1, v/v). The working standard solutions were prepared by further diluting the stock solutions in CHCl_3_:MeOH (2:1, v/v) to achieve concentrations of 250 ng mL^−1^ and 3.5 μg mL^−1^.

Calibration curves using 1-hexadecyl-2-(9Z-octadecenoyl)-sn-glycero-3-phosphocholine (PC(16:0e/18:1(9Z))), 1-(1Z-octadecenyl)-2-(9Z-octadecenoyl)-sn-glycero-3-phosphocholine (PC(18:0p/18:1(9Z))), 1-octadecanoyl-sn-glycero-3-phosphocholine (LPC(18:0)), 1-(1Z-octadecenyl)-2-docosahexaenoyl-*sn*-glycero-3-phosphocholine (PC(18:0p/22:6)) and 1-stearoyl-2-linoleoyl-*sn*-glycerol (DG(18:0/20:4)) from Avanti Polar Lipids Inc., 1-Palmitoyl-2-Hydroxy-sn-Glycero-3-Phosphatidylcholine (LPC(16:0)) from Larodan, and 1,2,3-Triheptadecanoylglycerol (TG(17:0/17:0/17:0)) and 3β-Hydroxy-5-cholestene 3-linoleate (ChoE(18:2)) from Sigma-Aldrich were prepared to the following concentration levels: 100, 500, 1000, 1500, 2000 and 2500 ng mL^−1^ (in CHCl_3_:MeOH, 2:1, v/v) including 250 ng mL^−1^ of each QC standard.

A total of 428 plasma samples were extracted in randomised order using a modified version of the Folch procedure: 10 μL of 0.9% NaCl, 40 μL of CHCl_3_:MeOH (2:1, v/v) and 80 μL of the 3.5 μg mL^−1^ working standards solution were added to 10 μL of each plasma sample. The samples were vortex mixed and incubated on ice for 30 min after which they were centrifuged (9400 × *g*, 3 min, 4 °C). From the lower layer of each sample, 60 μL was transferred to a glass vial with an insert and 60 μL of CHCl_3_:MeOH (2:1, v/v) and added to each sample. The samples were re-randomised and stored at −80 °C until analysis on the UHPLC-QTOF-MS system.

The UHPLC system used was a 1290 Infinity system from Agilent Technologies (Santa Clara, California, USA). The system was equipped with a multisampler (maintained at 10 °C) using 10% DCM in MeOH and ACN:MeOH:IPA:H_2_O (1:1:1:1, v/v/v/v) + 0.1% HCOOH as needle wash solutions after each injection for 7.5 s each, a quaternary solvent manager and a column thermostat (maintained at 50 °C). Separations were performed on an ACQUITY UPLC® BEH C18 column (2.1 mm × 100 mm, particle size 1.7 μm) by Waters (Milford, USA). The flow rate was 0.4 mL min^−1^ and the injection volume was 1 μL. H_2_O + 1% NH_4_Ac (1 M) + 0.1% HCOOH (A) and ACN:IPA (1:1, v/v) + 1% NH_4_Ac + 0.1% HCOOH (B) were used as the mobile phases for the gradient elution. The gradient was as follows: from 0 to 2 min. 35-80% B, from 2 to 7 min. 80–100% B and from 7 to 14 min 100% B. Each run was followed by a 7 min re-equilibration period under the initial conditions (35% B).

The mass spectrometer coupled to the UHPLC was a 6550 iFunnel QTOF-MS from Agilent Technologies interfaced with a dual jet stream electrospray (dual ESI) ion source. Nitrogen generated by a nitrogen generator (PEAK Scientific, Scotland, UK) was used as the nebulising gas at a pressure of 21 psi, as the drying gas at a flow rate of 14 L min^−1^ (at 193 °C) and as the sheath gas at a flow rate of 11 L min^−1^ (at 379 °C). Pure nitrogen (6.0) from Praxair (Fredericia, Denmark) was used as the collision gas. The capillary voltage and the nozzle voltage were kept at 3643 V and 1500 V, respectively. The reference mass solution including ions at *m/z* 121.0509 and 922.0098 was prepared according to instructions by Agilent and was introduced to the mass spectrometer through the other nebuliser in the dual ESI ion source using a separate Agilent series 1290 isocratic pump at a constant flow rate of 4 mL min^−1^ (split to 1:100 before the nebuliser). The acquisition mass range was *m/z* 100–1700 and the instrument was run in extended dynamic range mode with an approximate resolution of 30,000 FWHM measured at *m/z* 1521.9715 (which is included in the tune mixture) during calibration of the instrument. MassHunter B.06.01 software (Agilent) was used for data acquisition.

### Data pre-processing

**Figure 2:**
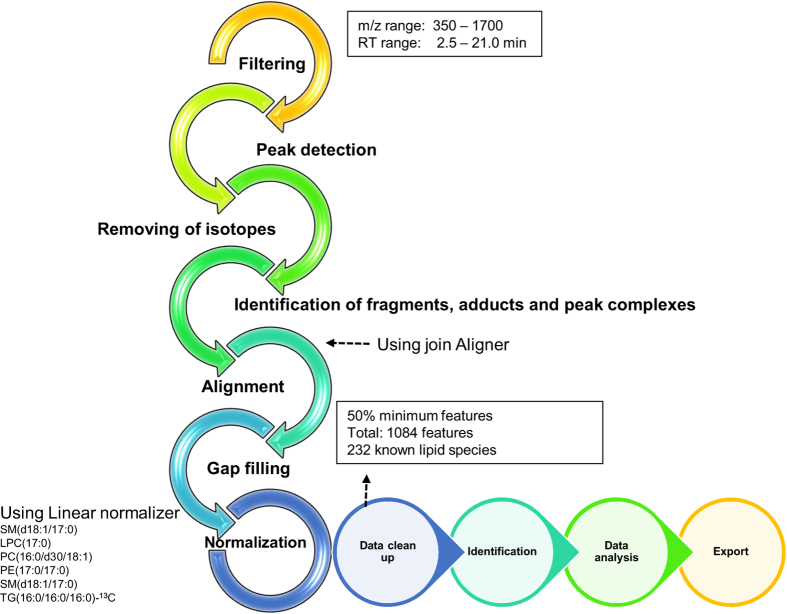
The typical workflow for processing mass spectrometry data using MZmine 2. The workflow includes raw file import, filtering/smoothing, detection of peaks, peak list de-isotoping, alignment, gap filling, and integration of peaks, normalisation, and, finally, peak/feature identification.

The data pre-processing was performed using MZmine 2.18.2^15^. Here, we have adhered to the data processing steps as suggested by the metabolomics standards initiative^16^. The typical pre-processing workflow includes raw file import, filtering/smoothing, detection of peaks, peak list de-isotoping, alignment, gap filling, integration of peaks, normalisation and, finally, peak/feature identification (Fig. 2, and Supplementary Table S1). The following steps were applied in the processing: (1) crop filtering with a *m/z* range of 350–1700 m/z and a retention time (RT) range of 2.5 to 21.0 min, (2) mass detection with a noise level of 750, (3) chromatogram builder with a minimum time span of 0.08 min, minimum height of 2250 and a *m/z* tolerance of 0.006 *m/z* or 10.0 ppm, (4) Chromatogram deconvolution using the local minimum search algorithm with a 70% chromatographic threshold, 0.05 min minimum RT range, 5% minimum relative height, 2250 minimum absolute height, a minimum ration of peak top/edge of 1 and a peak duration range of 0.08 to 5.0, (5) isotopic peak grouper with a *m/z* tolerance of 5.0 ppm, RT tolerance of 0.05 min, maximum charge of 2 and with the most intense isotope set as the representative isotope, (6) peak filter with minimum 12 data points, a FWHM between 0.0 and 0.2, tailing factor between 0.45 and 2.22 and asymmetry factor between 0.40 and 2.50, (7) peak list row filter keeping only peak with a minimum of 1 peak in a row, (8) join aligner with a *m/z* tolerance of 0.006 or 10.0 ppm and a weight for of 2, a RT tolerance of 0.1 min and a weight of 1 and with no requirement of charge state or ID and no comparison of isotope pattern, (9) peak list row filter with a minimum of 53 peak in a row (=10% of the samples), (10) duplicate peak filter with a *m/z* tolerance of 0.006 m/z or 10.0 ppm and a RT tolerance of 0.1 min, (11) gap filling using the same RT and *m/z* range gap filler algorithm with an *m/z* tolerance of 0.006 m/z or 10.0 ppm, (12) peak filter using the same parameters as under step 6, (13) peak list row filter with a minimum of 265 peaks in a row (=50% of the samples), enabling detection of 1084 features (14) identification of lipids using a custom database search (232 identified out of 1084 features) with an *m/z* tolerance of 0.006 m/z or 10.0 ppm and a RT tolerance of 0.1 min, (15) normalisation using lipid-class-specific internal standards (PE (17:0/17:0), SM (d18:1/17:0), Cer (d18:1/17:0), LPC (17:0), TG (16:0/16:0/16:0)-13C3 and PC (16:0/d30/18:1)) using in-house developed R-script, and (16) data imputation of missing values with the half of the minimum value for each lipid.

### Code availability

All the pre-processing analyses were performed using the publicly-available software package MZmine 2.18.2 and with parameters as described in Supplementary Table S1.

## Data records

The raw data files in .mzML format are deposited in the MetaboLights repository (Data Citation 1). Additionally, the deposited data contains the pre-processed lipidomic data as obtained from MZmine data processing, including the identified lipids. The associated data were captured using the ISA-creator package available from MetaboLights. This manuscript describes the samples, data collection, processing steps and overall study design.

### Ethical approval and informed consent

The ethics and research committees of both the participating university and hospital at University of Tampere, Tampere Finland, approved the study protocol. The study was conducted according to the guidelines in the Declaration of Helsinki. All families provided written informed consent for participation in the study.

## Technical validation

This study followed the best practices of analytical methodologies for the global profiling of lipids in the plasma sample as described by Hyotylainen *et al.*^17,18^. For quality monitoring, QC samples (pooled plasma samples), blank samples, pure standards (standards in solvent) were run at regular intervals after every 4–16 study samples. To assure data quality, relative standard deviations (%RSDs) were calculated for the retention times and the peak areas for each QC standard in the QC samples throughout the sample set. Overall, these results show a reproducible method. For example, pooled plasma samples analysed throughout the sample set show average %RSDs for the four batches of samples of less than 0.2 and 15.4% (normalised using PC(16:0/d30/18:1)) for the retention times and peak areas, respectively. The %RSDs for the rest of the QC samples were in the same range as for the pooled plasma samples.

**Figure 3:**
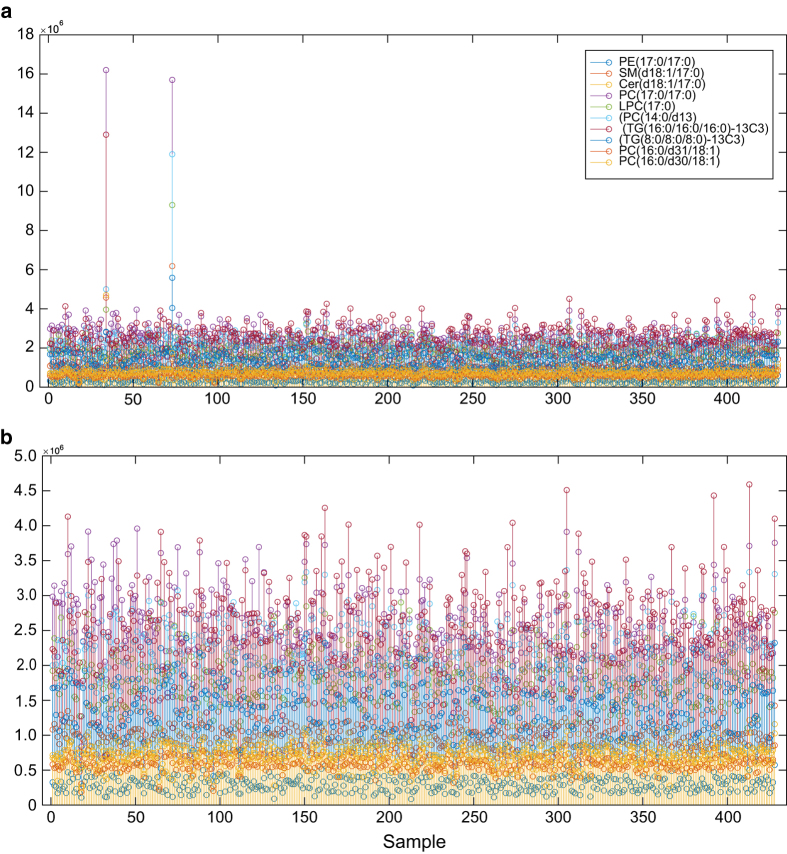
Quality control of UHPLC-QTOFMS run evaluated using peak areas from nine internal standard compounds. (**a**) Plot of peak areas with 430 samples. (**b**) Plot of peak areas of 428 samples, *i.e.* after removal of 2 outlier samples. Here, each color represents the peak area of the given internal standard PE(17:0/17:0), SM(d18:1/17:0), Cer (d18:1/17:0), PC(17:0/17:0), LPC(17:0), PC(14:0/d13), TG(16:0/16:0/16:0)-13C3, TG(8:0/8:0/8:0)-13C3 and PC(16:0/d31/18:1).

To assess experimental error, nine internal standards were spiked into the plasma samples during sample preparation. The plot of peak areas from nine QC standards (Fig. 3a) clearly shows that two samples had eccentric internal standard profiles. This was traced to an error during sample preparation, wherein the QC standard was added two these two samples twice. Therefore, these two outlier samples were not included in downstream analysis (and not included in the data descriptor). The representative quality plots after removal of aberrant samples (Fig. 3b) demonstrates that the peak areas of the QC standards have very low variability between the measured samples. This indicates that use of proper internal standards and careful follow-up of the response of these internal standards critically reduces the chances of unexpected variation, which may arise in LC-MS analysis. Based on this, we conclude that the samples in this data descriptor were successfully analysed following generally accepted analytical quality guidelines.

During LC-MS analyses, it is also highly important to keep track of blank samples inserted into the sample sequence. This is done to detect possible contamination/interference, which affect the actual sample data. We analysed 293 blank or solvent blank samples throughout this sample set. Solvent blank samples (consisting of a mixture of CHCl_3_: MeOH, 2:1, v/v) were analysed after every 4^th^ sample and blank samples (the same solvent mixture, which underwent the normal sample preparation procedure except for the addition of internal standards) after every 16^th^ sample. Additionally, a set of QC samples (186 samples) were analysed at the same frequency as the blank samples (*i.e.* after every 16^th^ sample). This set of QC samples consisted of: (1) extracted and non-extracted standard samples (the chosen QC standards in solvent), (2) a pooled plasma sample originating from a larger plasma pool from actual diabetes patients for following up on long-term method performance and to facilitate batch correction if needed, and (3) a Standard Reference Material 1950 sample from the National Institute of Standards and Technology (NIST) that serve as community-wide benchmarks for intra- and inter-laboratory QC and method validation^19^.

**Figure 4:**
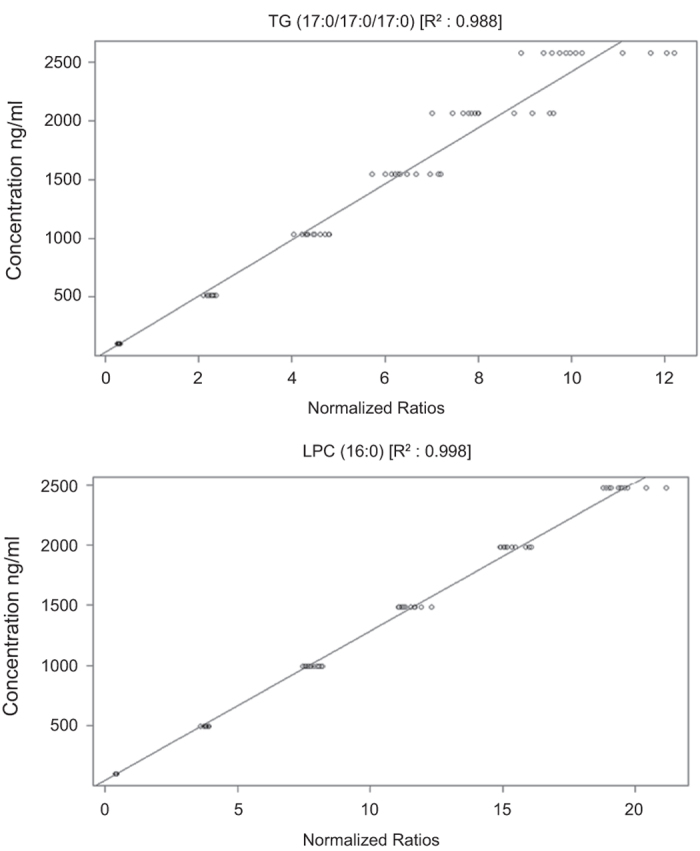
Representative global linear models. Six different concentrations of lipid class-specific standards TG (17:0/17:0/17:0), and LPC (16:0) were used to generate the calibration plot.

Next, for quantitative analysis, calibration curves of each analyte must be generated. For global lipidomics analyses this is, however, logistically and practically impossible due to the high number of analytes present in the biological sample matrix. To overcome this limitation, lipid-class-specific compounds were used for creating a semi-quantitative method including six different concentrations of the lipid class specific standards PC (16:0e/18:1(9Z)), TG (17:0/17:0/17:0), LPC (18:0), DG (18:0/20:4) and CE (18:2). The acceptance of LC-MS data usually relies on QC samples being successfully quantified within pre-defined limits. Achievement of this standard is evaluated statistically (global linear models) based on the value for the coefficient of determination (*R*^*2*^). Figure 4 shows coefficients of determination higher than 0.9 for TG (17:0/17:0/17:0) and LPC(16:0) at six different concentrations using a global linear model with a 1/x weighting. The other standards show similar values (i.e. *R*^*2*^ > 0.9). This statistical analysis consequently ensured the reliability of results presented in this data descriptor.

Furthermore, the assurances provided by the QC methods herein derive from analytical reproducibility, within or between batches of the LC-MS data. These inter- and intra-batch variations can be assessed and corrected using the QC samples. The principal component analysis (PCA) score plot (Supplementary Figure S1) highlights the aforementioned reproducibility across all batches obtained from the pre-processed dataset in this data descriptor. From this score plot, it is apparent that there are no patterns or any clustering that correlated with the batch run during LC-MS. Together, the dataset in this data descriptor reflects robustness of lipid measurement over time, which, in turn, indicates that data analysis performed using this dataset will not be affected by systematic error arising due to instrumental factors.

## Usage notes

The pre-processed data are available from MetaboLights (Data Citation 1). The detail parameters for data pre-processing are available in supplementary material (Supplementary Table S1). The pre-processed data were normalised using the nine different class-specific internal standards. The class-specific were based on PE (17:0/17:0), SM (d18:1/17:0), Cer (d18:1/17:0), LPC (17:0), TG (16:0/16:0/16:0)-13C3 and PC (16:0/d30/18:1), while others were based on retention time; LPC(17:0) for <= 6.0 min elutions, PC(16:0/d30/18:1) for >6.0, <9.0 min elutions and TG(16:0/16:0/16:0)-13C3 for > = 9.0 min elutions. In addition, the zero values in the lipidomics dataset were imputed with half of that row’s minimum value for each lipid (the code for this imputation were written in-house using R, and are available on request). Due to the non-normal distribution of data, it is recommended that the data are log-transformed before analysis (log-transformation performed in MZmine 2.18.2). Other required guidelines for data-preprocessing are available from the MZmine website (http://mzmine.github.io/documentation.html) and authors can be contacted for further help if required.

## Additional information

**How to cite this article**: Lamichhane, S. *et al*. A longitudinal plasma lipidomics dataset from children who developed islet autoimmunity and type 1 diabetes. *Sci. Data*. 5:180250 doi: 10.1038/sdata.2018.250 (2018).

**Publisher’s note**: Springer Nature remains neutral with regard to jurisdictional claims in published maps and institutional affiliations.

## Supplementary Material



Supplementary Information

## Figures and Tables

**Table 1:** Anthropometric characteristics of study population.

	**PT1D (n = 40)**	**P1Ab (n = 40)**	**CTR(n = 40)**
Gender (girls, boys)	(14, 26)	(14, 26)	(14, 26)
Age at time of diagnosis (years) (mean ± SD)	4.75 ± 2.94	NA	NA
Age at time of first seroconversion (years) (mean ± SD)	1.34 ± 0.58	3.05 ± 2.50	NA
