# Supplementary material for: A longitudinal plasma lipidomics dataset from children who developed islet autoimmunity and type 1 diabetes
Source: Sci Data. 2018 Nov 13;5:180250. doi: 10.1038/sdata.2018.250 (PMC6233478; doi:10.1038/sdata.2018.250)
Supplement: Supplementary Information [file sdata2018250-s2.docx]

# A longitudinal plasma lipidomics dataset from children who developed islet autoimmunity and type 1 diabetes.

**Supplementary information**

Supplementary Information: Contents

1. Supplementary Table S1 …………………………………………………..…Page 2-4
2. Supplementary Figure S1………………………………………………..Page 5

Supplementary Table S1

| SN | Data Pre-Processing parameters | Values in MZmine 2.18.2 |
| --- | --- | --- |
| 1 | **Crop filter** | m/z range 350 – 1700 RT range 2.5 – 21.0 min |
| 2 | **Mass Detection** | Mass detector Centroid MS level 1 Noise level 750 |
| 3 | **Chromatogram Builder** | Min. time span 0.08 min Min. height 2250 (3x noise level) m/z tolerance 0.006 m/z or 10.0 ppm |
| 4 | **Chromatogram Deconvolution** | Algorithm Local minimum search Chromatographic threshold 70% Minimum in RT range 0.05 min Minimum relative height 5% Minimum absolute height 2250 (3x noise level) Min ration of peak top/edge 1 Peak duration range 0.08 - 5.0 |
| **5.** | **Isotopic Peak Grouper** | m/z tolerance 0 m/z or 5.0 ppm RT tolerance 0.05 min (3.0 sec) Monotonic shape FALSE Maximum charge 2 Representative isotope Most intense |
| **6.** | **Peak Filter** | # data points Min. 12 FWHM 0.00 – 0.20 Tailing factor 0.45 – 2.22 Asymmetry factor 0.40 – 2.50 |
| **7.** | **Peak List Row Filter** | Minimum peaks in a row 1 |
| **8.** | **Join Aligner** | m/z tolerance 0.006 m/z or 10.0 ppm Weight for m/z 2 RT tolerance 0.1 min Weight for RT 1 Require same charge state FALSE Require same ID FALSE Compare isotope pattern FALSE |
| **9.** | **Peak List Row Filter** | (Conservative) Minimum peaks in a row 10% (53 samples) |
| **10.** | **Duplicate peak filter** | m/z tolerance 0.006 m/z or 10.0 ppm RT tolerance 0.1 min |
| **11.** | **Gap Filling – Peak Finder** | Intensity tolerance 10% m/z tolerance 0.006 m/z or 10.0 ppm RT tolerance 0.1 min RT correction FALSE |
| **12.** | **Peak Filter** | Data points Min. 12  FWHM 0.00 – 0.20  Tailing factor 0.45 – 2.22 Asymmetry factor 0.40 – 2.50 |
| **13.** | **Peak List Row Filter** | (Strict) Minimum peaks in a row 50% (265 samples) |
| **14.** | **Identification – Custom Database Search** | m/z tolerance 0.006 m/z or 10.0 ppm  RT tolerance 0.1 min  MS Library version 2016-01-012 |
| **15.** | **Targeted detection of standards and calibrants** | Intensity tolerance 20 % Noise level 2250 m/z tolerance 0.006 m/z or 10.0 ppm RT tolerance 0.1 min |
| **16.** | **Normalisation** | *Class specific:*  CE SM(d18:1/17:0)  LPC LPC(17:0)  PC PC(16:0/d30/18:1) PE PE(17:0/17:0)  SM SM(d18:1/17:0)  TG TG(16:0/16:0/16:0)-13C3  *All others – RT based:* <= 6.0 min LPC(17:0) > 6.0, <9.0 min PC(16:0/d30/18:1)  >= 9.0 min TG(16:0/16:0/16:0)-13C3 |
| **17.** | **Calibration**  **Linear model with 1/x weight.** | *Class specific:*  CE CE(18:2) LPC LPC(16:0) PE PC(16:0e/18:1(9Z))  PC PC(16:0e/18:1(9Z))  SM CE(18:2)  TG TG(17:0/17:0/17:0)  *All others – RT based:* <= 6.0 min LPC(16:0) > 6.0, <9.0 min PC(16:0e/18:1(9Z))  >= 9.0 min TG(17:0/17:0/17:0)  Concentrations – 100 ng/ml sample: CE(18:2) 103.6 ng/ml LPC(16:0) 99.1 ng/ml PC(16:0e/18: 1(9Z)) 100 ng/ml TG(17:0/17:0/17:0) 103.2 ng/ml |
| **18.** | **Data clean-up**  Min # features in a batch row: 50% |  |

**Table S1: Data pre-processing parameters values in MZmine 2.18.2**

Supplementary Figure S1


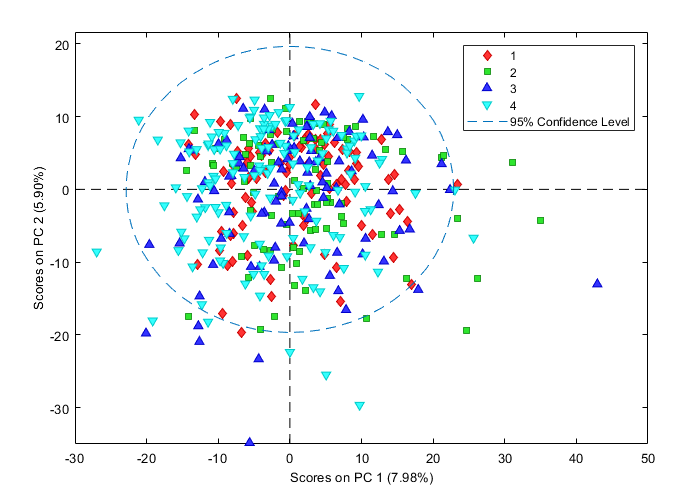


**Figure S1:** Principal Component Analysis (PCA). These PCA scores represent the preprocessed lipidomics dataset from 428 plasma samples ran across 4 batches. Here each sample is represented by a point and each color represent a single batch. No pattern or any clustering is seen in the PCA score plot.
